# A Case of Mesial Temporal Lobe Sclerosis Following Temporal Bone Encephalocele Repair for Medically Refractory Seizures

**DOI:** 10.7759/cureus.3623

**Published:** 2018-11-22

**Authors:** Helena Wichova, Matthew Shew, Sameer Alvi, James Lin

**Affiliations:** 1 Otolaryngology, University of Kansas Medical Center, Kansas City, USA; 2 Otolaryngology, Rush University Medical Center, Chicago, USA

**Keywords:** encephalocele, encephalocele, mesial temporal lobe sclerosis, mastoidectomy, seizure disorder, seizure disorder

## Abstract

The aim of this report is to present a case of mesial temporal lobe sclerosis (MTS) causing medically refractory seizures, which was initially disguised as temporal lobe encephalocele secondary to prior otologic surgery. Temporal lobe encephaloceles are characterized by a defect within the middle cranial fossa that results in the abnormal communication of the meninges into the pneumatized skull base. After the temporal lobe encephalocele repair, the patient continued to have seizures and was subsequently diagnosed with mesial temporal lobe sclerosis. Imaging revealed the serial progression of hippocampal atrophy and loss of internal architecture. Differentiation between mesial temporal sclerosis and encephalocele as the underlying epileptic etiology is critical. While repairing encephaloceles is necessary to address other potential sequelae, patients with mesial temporal lobe sclerosis will require additional interventions.

## Introduction

Temporal lobe encephaloceles (TLEs) are characterized by a defect within the middle cranial fossa that results in the abnormal communication of meninges into the pneumatized skull base. TLEs can be iatrogenic, idiopathic, congenital, or secondary to destructive processes such as trauma, cholesteatoma, infection, or tumors [1-2]. Symptoms can be variable and include cerebrospinal fluid (CSF) fistulae, conductive hearing loss, headaches, meningitis, and seizures [3]. Although rare, medically refractory seizures can be seen in up to 2% of patients with TLE [2]. Nearly 100% success rates on the resolution of seizures are reported following temporal lobe encephalocele repair [1-2,4]. We report an atypical imaging case of a patient who underwent TLE repair for medically refractory seizures. The patient continued to have seizures following repair and, upon further imaging, was diagnosed with mesial temporal lobe sclerosis (MTS). MTS is due to sclerosis of the hippocampus, which is seen as gliosis and volume loss on imaging [5]. With MTS, electroencephalography (EEG) activity lateralizes to the temporal lobe on the ipsilateral side [6].

## Case presentation

**Figure 1:**
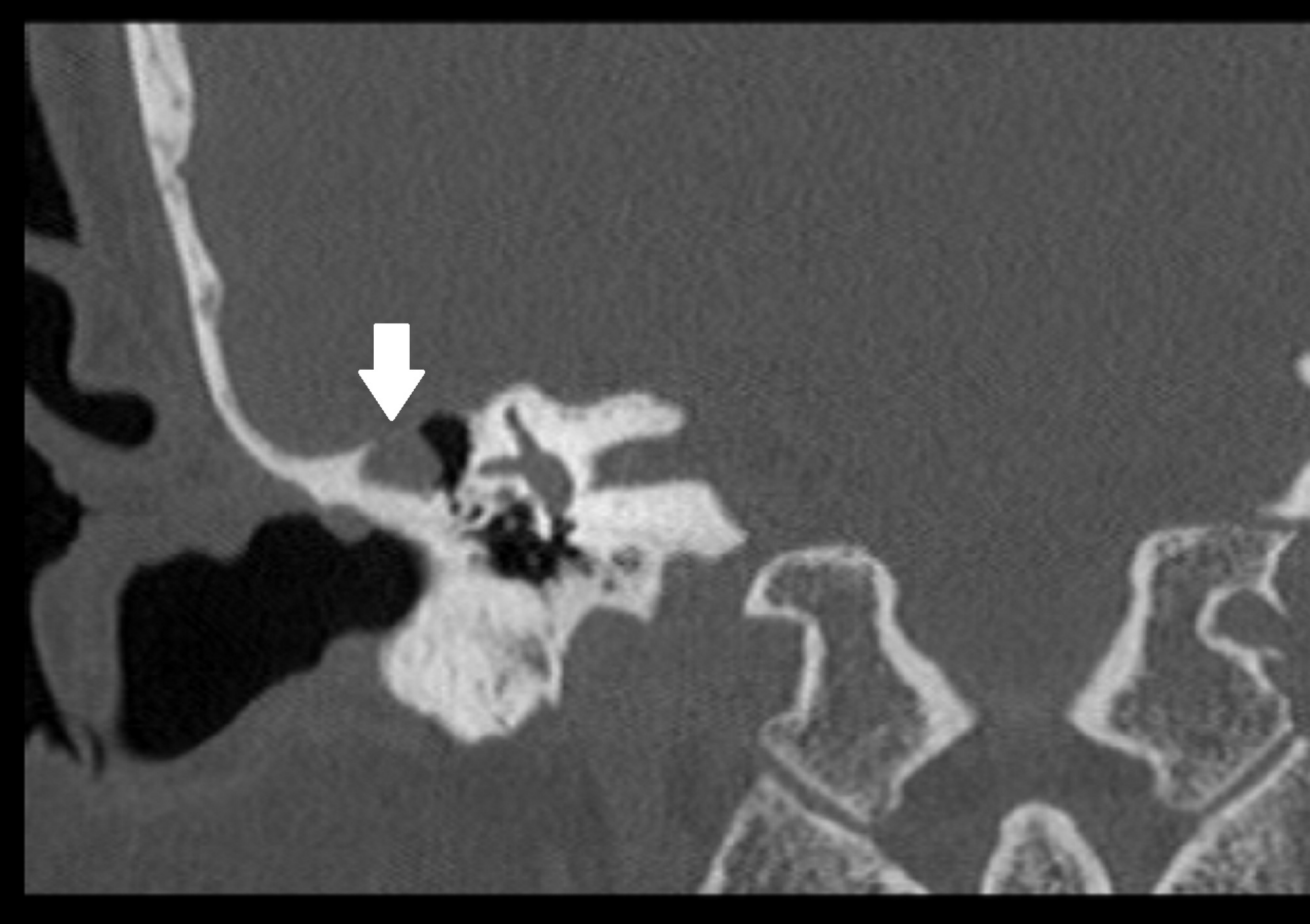
High-resolution CT scan highlighting the temporal bone defect and the associated encephalocele CT: computed tomography

**Figure 2:**
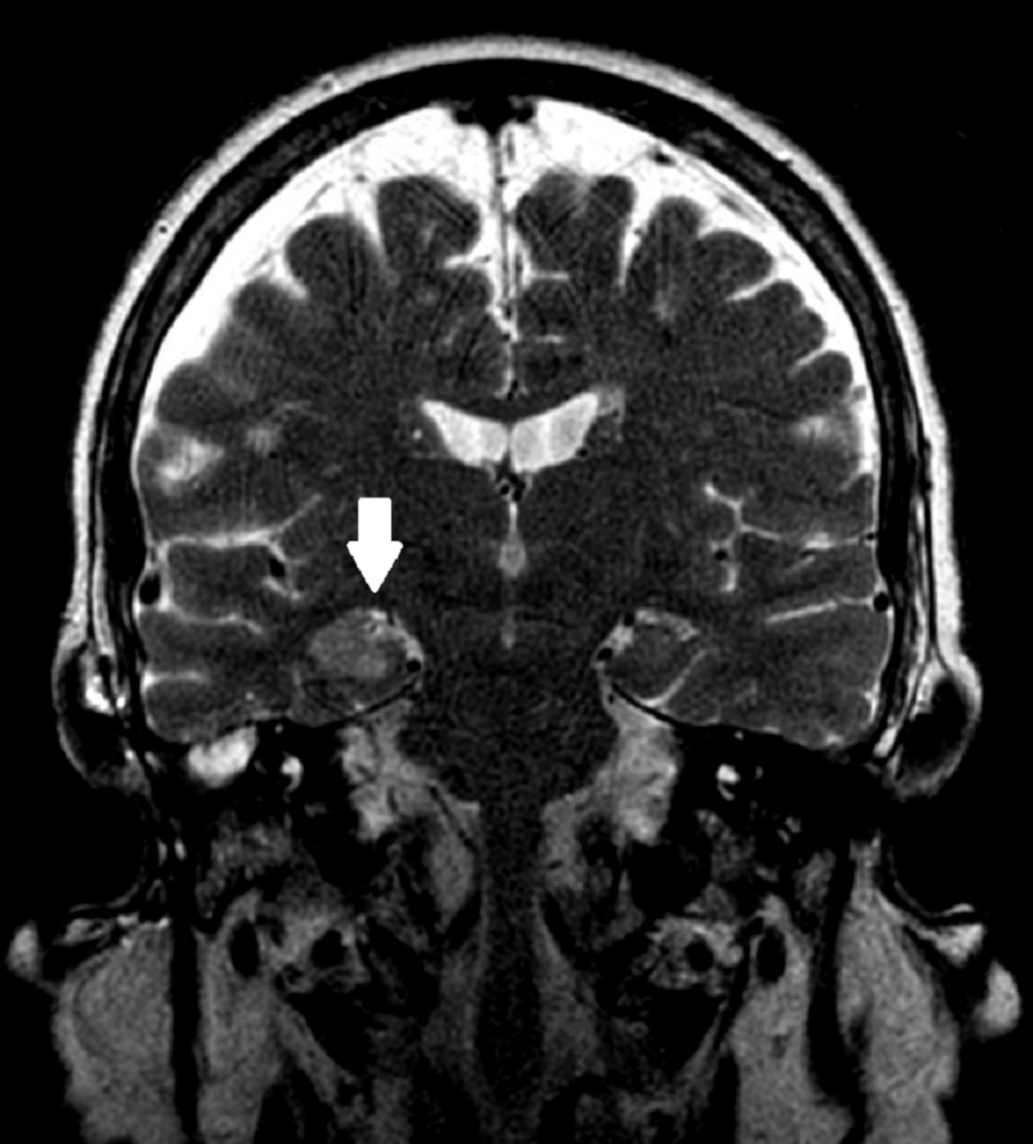
1.5 Tesla MRI protocol for epilepsy on initial presentation T2-weighted image: Arrow points to increased edema and flair within the right mesial temporal lobe MRI: magnetic resonance imaging

An 80-year-old female was referred with a six-month history of medically refractory seizures and evidence of a right tegmen dehiscence and encephalocele. Her medical history was significant for chronic otitis media, with a history of a right-sided tympanomastoidectomy 20 years prior and ongoing follow-up for chronic eustachian tube dysfunction. Her symptoms included daily episodic paresthesias with phantosmia and a right-sided severe to profound mixed hearing loss. She denied CSF rhinorrhea, otorrhea, or other symptoms. Seizure workup included video EEG confirming right temporal lobe epilepsy as a cause of her paresthesias and phantosmia. Computed tomography (CT) and magnetic resonance imaging (MRI) demonstrated a right temporal lobe encephalocele. In addition to the TLE, MRI demonstrated increased edema and flair signal within the right mesial temporal lobe (Figures 1-2).

**Figure 3:**
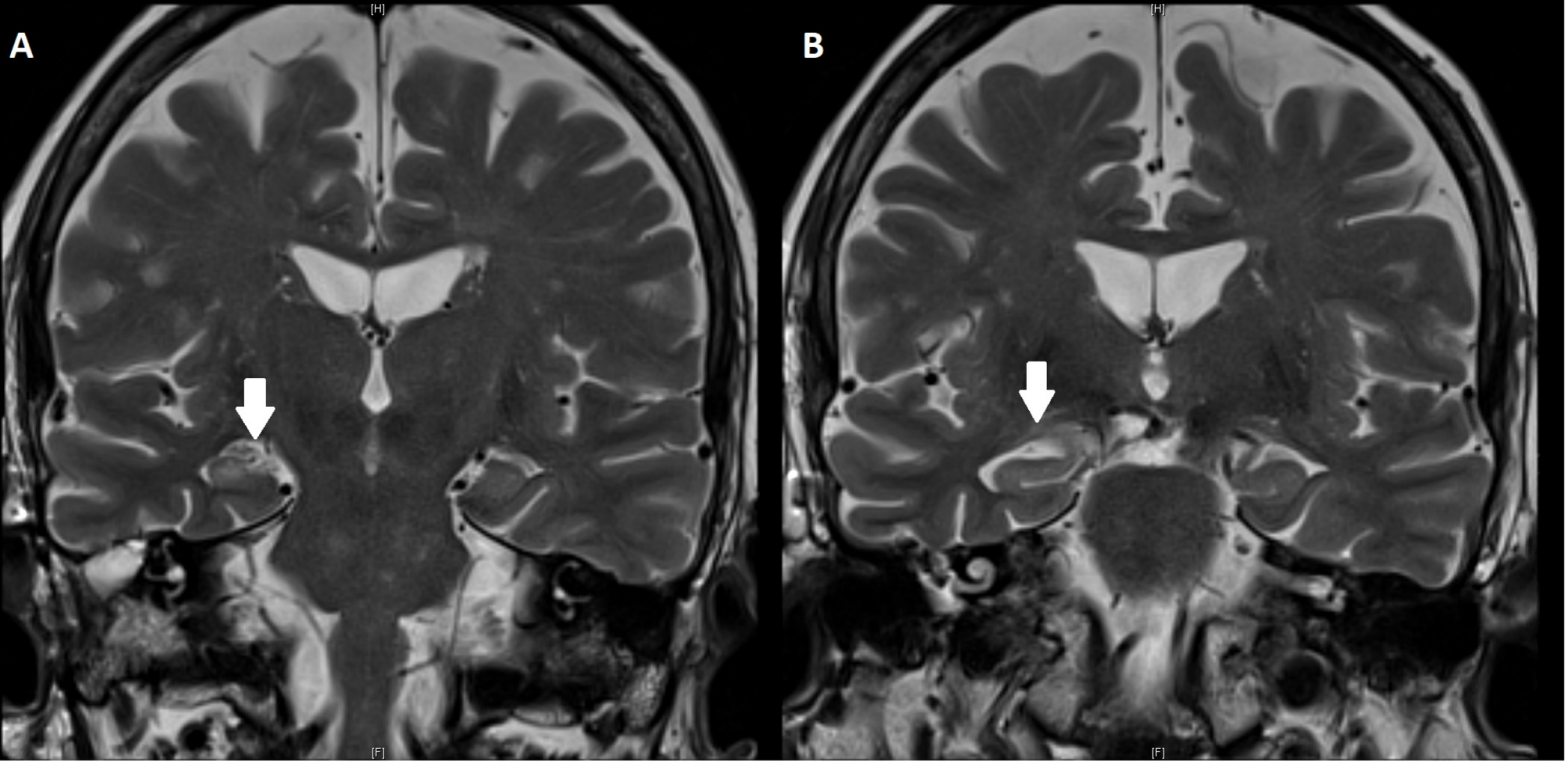
3 Tesla MRI at four months postoperative follow-up A) Reduction of hippocampal edema; B) Increased signal and loss of internal architecture of the hippocampus diagnostic for MTS MRI: magnetic resonance imaging; MTS: mesial temporal lobe sclerosis

She was discussed at a multidisciplinary skull base conference with the decision to undergo a combined mastoid-middle cranial fossa encephalocele repair. Informed consent was obtained prior to proceeding with surgery. Intraoperatively, a 1 x 1 cm tegmen defect with the herniation of glial tissue into the mastoid was repaired with partial resection and an Onlay dural substitute (Lyoplant, Aesculap, Tuttlingen, Germany). Her postoperative course was uncomplicated. Immediately, she noted an improvement in her seizure frequency and duration; however, her seizures did not fully resolve. A repeat 3T epilepsy protocol MRI demonstrated further hippocampal atrophy, increased flair within the right hippocampus, and the loss of gray-white differentiation in the anterior temporal lobe, diagnosing mesial temporal lobe sclerosis (Figures 3A-3B). On retrospective neuroradiology review, the progression of decreased hippocampal volume was noted on MRIs leading up to surgery. She is currently undergoing workup for resection of the temporal epileptically focal lesion.

## Discussion

Over 80% of medically refractory seizures arise from the temporal lobe [4,7]. MTS, also known as hippocampal sclerosis, is believed to be the leading associated pathology [7]. The cause and effect relationship between temporal lobe epileptic foci and MTS remains controversial [7]. On a cellular level, hypothesized causative factors include hypoxia secondary to status epilepticus, the neurotoxic effects of excessive glutamate production, and hypoglycemia [8]. The utilization of 3 Tesla seizure-protocol MRI is becoming prevalent for diagnosis with specificity and sensitivity approaching 90% as compared to 21% with standard 1.5 Tesla MRI [7]. Radiographic findings include hippocampal atrophy, increased T2 and fluid-attenuated inversion recovery (FLAIR) signal, and loss of internal architecture within the hippocampus [7]. The utility of MRI for the diagnosis of MTS in the postictal state, as in our case, can be challenging. It can be difficult to differentiate resolving hippocampus edema from the loss of internal architecture within the hippocampus in MTS. Recently, magnetic resonance techniques, including volumetric analysis, perfusion, diffusion-weighted, and spectroscopy, have been used to supplement the diagnosis in equivocal cases [7,9-10]. While EEG is the standard of care in seizure workup for temporal lobe epilepsy, it is clear that various MRI modalities are also useful in identifying mesial versus lateral epileptogenic foci that are amenable to intervention [10]. Once a diagnosis is established, anterior temporal lobectomy or selective amygdalohippocampectomy have been shown to be 75%-90% effective for patients with MTS [7].

Differentiation between MTS and encephalocele as the underlying epileptic etiology is critical. While repairing encephaloceles, it is necessary to address other potential sequelae. Patients with MTS may continue to have seizures, as demonstrated in our case. On retrospective review with neuroradiology, sequential MRI findings prior to surgical intervention were suggestive but not diagnostic for MTS. Serial MRIs over a four-month period showed a persistent flair signal and a reduction in the size of the ipsilateral hippocampus. The initial edema and pattern of volume loss within the hippocampus made it difficult to distinguish the resolution of the postictal state versus the development of MTS.

## Conclusions

The recognition of MTS as a possible source of an underlying epileptic etiology is critical. While repairing TLEs, it is necessary to address other potential sequelae, as patients with MTS will require additional interventions. Therefore, we advocate that in patients referred for TLE and seizures, careful MRI evaluation of the ipsi- and contralateral hippocampus, with attention to volumetric analysis, is essential. Any size differences within the hippocampus or radiographic features suggestive of MTS should instigate a discussion among treating teams.
